# Comparison of pressure vs. volume controlled ventilation on oxygenation parameters of obese patients undergoing laparoscopic cholecystectomy

**DOI:** 10.12669/pjms.335.13316

**Published:** 2017

**Authors:** Reza Movassagi, Majid Montazer, Ata Mahmoodpoor, Vahid Fattahi, Afshin Iranpour, Sarvin Sanaie

**Affiliations:** 1 Reza Movassagi, Assistant Professor, Department of Anesthesiology, Faculty of Medicine, Tabriz University of Medical Sciences, Golgasht, Iran; 2 Majid Montazer, Assistant Professor, Evidence Base Medicine Research Center, Tabriz University of Medical Sciences, Golgasht, Iran; 3 Prof. Ata Mahmoodpoor, Department of Anesthesiology, Fellowship of Critical Care Medicine, Faculty of Medicine, Tabriz University of Medical Sciences, Golgasht, Iran; 4 Vahid Fattahi, Anesthesiologist, Anesthesiology Research Team, Tabriz University of Medical Sciences, Golgasht, Iran; 5 Afshin Iranpour, Anesthesiologist, Department of Anesthesiology, Al Zahra Hospital, Dubai, UAE; 6 Sarvin Sanaie, Assistant Professor, Tuberculosis and Lung Disease Research Center, Tabriz University of Medical Sciences, Golgasht, Iran

**Keywords:** Laparoscopy, Obese, Oxygenation, Pressure controlled ventilation, Volume controlled ventilation

## Abstract

**Background & Objective:**

There is no special guideline for the best ventilation mode during laparoscopic anesthesia in obese patients and there are too many studies with different controversial points. The aim of this study was to compare the effect of pressure controlled ventilation (PCV) vs. volume controlled ventilation (VCV) on respiratory and oxygenation parameters in patients undergoing laparoscopic cholecystectomy.

**Methods:**

Seventy patients with 30 <BMI<40 and ASA physical status I-II were studied in this randomized prospective trial. Anesthesia was started with VCV and after creation of pneumoperitoneum; the patients were randomized into PCV or VCV groups. Ventilation parameters were adjusted to a CO2 target of 35-40 mmHg. Hemodynamic and oxygenation parameters and respiratory parameters like plateau, mean airway and peak pressure were recorded for all patients during the study.

**Results:**

Patients in VCV group needed higher tidal volume and respiratory rate to maintain target CO_2_ in 35 and 55 minutes after the study. Plateau pressure and mean airway pressure in two groups didn’t have significant difference between two groups but peak airway pressure in 35 and 55 minutes after pneumoperitoneum was significantly higher in VCV group than PCV group. There were no significant differences between two groups regarding PO_2_, PCO_2_ and pH, except 35 and 55 minutes after pneumoperitoneum. In mentioned times, patients in PCV group had significantly higher PO_2_ levels compared to VCV group.

**Conclusion:**

Despite some beneficial effects regarding plateau, mean airway pressure and oxygenation parameters with PCV, there was no significant clinical difference between PCV and VCV in obese patients undergoing laparoscopic cholecystectomy.

## INTRODUCTION

Optimization of intraoperative mechanical ventilation can decrease the incidence of pulmonary postoperative complication and improve outcome especially in obese patients.^1^ Volume controlled ventilation(VCV) has been the most frequently used mode of ventilation during anesthesia for a long time.^2^ This mode uses a constant flow to deliver tidal volume but can result in higher airway pressures especially during laparoscopic procedures due to pneumoperitoneum.^3^ Pneumoperitoneum results in decreased lung and chest wall compliance and reduced functional residual capacity which impairs alveolar ventilation and leads to ventilator induced lung injury.^4^,^5^

Pressure controlled ventilation (PCV) uses a decelerating flow which reaches the highest possible value at the beginning of inspiration, while having a preset pressure limitation with no minimum level for tidal volume. This has been attributed to the decelerating inspiratory flow delivery method, whereby high initial flow rates are delivered to quickly achieve and maintain the set inspiratory pressure followed by rapidly decelerating flow.^6^,^7^ This high initial rate of flow leads to a more rapid alveolar inflation and more homogenous distribution of ventilation to the lung and improving ventilation/perfusion mismatch.^8^ However, patients can receive inappropriate levels (low) of tidal volumes during pneumoperitoneum because of increased pressure. Using this mode results in low peak pressures and decreases the incidence of barotraumas especially in obese patients. Aydin et al. showed that VCV mode can provide better alveolar ventilation than PCV mode in patients undergoing laparoscopic cholecystectomy operations.^9^,^10^ Despite these potential benefits, use of PCV did not improve patients outcome in previous studies and there are some controversial reports.^9^,^10^

Choi et al showed that PCV offered a better compliance and lower peak pressures than VCV, but there were no advantages over VCV in hemodynamics.^11^ Based on the different results of previous trials on the type of mode used for ventilation during anesthesia, we performed this study to compare the effect of PCV vs. VCV on lung mechanics and oxygenation parameters in patients undergoing laparoscopic cholecystectomy.

## METHODS

After approval of ethics committee of Tabriz University of Medical Sciences, 80 obese patients who were candidate for laparoscopic cholecystectomy were enrolled in this prospective randomized trial from April 2014 till April 2016. (Registration number: IRCT201411203915N16). Referring to previous studies^12^, sample size of 40 patients for each group was calculated based on a mean difference of four in peak airway pressure between the PCV and VCV, with a population variance of (4)^2^, a two-sided alpha of 0.05 and a power of 80%. Informed consent was taken from all patients before enrollment. Inclusion criteria were 30<BMI<40, ASA physical status I- II, age of 18 to 60 years old and absence of severe pulmonary disease. Exclusion criteria were patients’ refusal, inability to perform extubation after anesthesia, inability to maintain stable mechanical ventilation setting for 30 minutes during anesthesia, inability to maintain appropriate ETCO_2_ during anesthesia and conversion to laparatomy.

Spirometry was performed before operation for each patient who needed it in order to exclude patients with moderate to severe chronic obstructive lung disease. If <70% of predicted value for pulmonary function test was shown, the patient was excluded. All patients were anesthetized based on the standard protocol and were randomized into two groups to receive PCV or VCV mode during operation. Standard protocol included ECG, noninvasive arterial pressure, pulseoximetry and end tidal CO_2_ monitoring. All patients received 0.5 mg intravenous midazolam for sedation before the induction of anesthesia. Induction was performed with 2 mg/kg of propofol, lidocaine 1 mg/kg and fentanyl 1.5 µg/kg and cisatracurium 0.15 mg/kg. Cisatracurium infusion was started to maintain muscle relaxation at < 2 twitches (train of four ratios) of the orbicular muscle of the eye. Bispectral index (BIS technology, Aspect Medical Systems, Meern, The Netherlands) was used to monitor the level of consciousness. After induction, intubation was performed and in all patients ventilation was performed with tidal volume of 8 ml/kg, inspiratory/expiratory ratio of 1/2 to maintain target ETCO_2_ of 35-40 mmHg. Patients were positioned head up (25 degree) after pneumoperitoneum with 10-12 mmHg of intra-abdominal pressure. Patients were randomized to one of the study groups 15 minutes after starting pneumoperitoneum.

In VCV group, ventilation was performed with 8 ml/kg and in order to keep ETCO_2_ in the range of 35-40 mmHg, tidal volume was increased incrementally by one ml/kg to 10 ml/kg each five minutes and respiratory rate were increased incrementally by two each five minutes to 25/min. If the target ETCO_2_ could not be achieved, patients were withdrawn from the study. In PCV group, pressure was set to target tidal volume of 8ml/kg and respiratory rate was optimized based on ETCO2 range of 35-40 mmHg. Respiratory rate was increased incrementally by two each five minutes to reach targeted CO_2_to maximum of 25 rates/minute and respiratory rate was decreased by 2 each 5 minutes if ETCO2 was less than the targeted value. If patients needed the pressure more than 35 mmHg or RR more than 25, they were excluded from the study in PCV group. In VCV group, patients were excluded if they needed more than 10 ml/kg of tidal volume or RR more than 25. PEEP was set on 5 cmH_2_O in both groups as the physiologic PEEP. Arterial blood gas analysis was performed at induction through an arterial line inserted in radial artery, 15 minutes after performing pneumoperitoneum and each 20 minutes after that until the end of operation. Oxygenation and lung dynamic parameters were noted for all patients during the study. Statistical analysis was performed with SPSS version 17 statistical package. Quantitative variables were analyzed with unpaired t test and categorical data were analyzed using Pearson’s chi square test. Repeated measure test was used for intra group data analysis and p value <0.05 was considered to be significant.

## RESULTS

**Fig.1:**
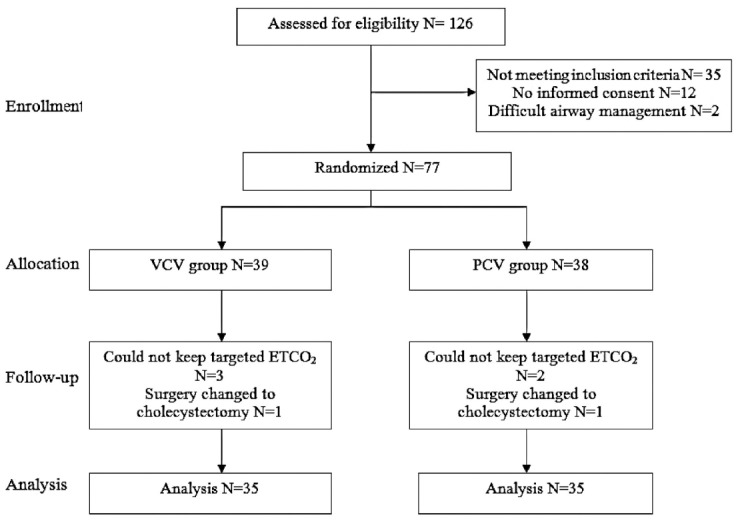
Study overview.

In this study, 126 patients were enrolled of whom 77 were randomized into two groups. Seven patients were later missed (two patients whose surgery was changed to open cholecystectomy and five patients in whom we couldn’t keep targeted ETCO2). Flow diagram of study is shown in Fig.1. Demographic characteristics and hemodynamic parameters of patients in two groups didn’t have significant differences (Table-I). Patients in VCV group needed statistically higher tidal volume and respiratory rate to keep target CO_2_ on 35 and 55 minutes after initiation of the study (Table-II). This means that patients in VCV group needed higher minute ventilation compared to PCV group on 35 and 55 minutes after initiation of the study. Plateau pressure and mean airway pressure didn’t have significant difference between two groups but peak airway pressure in 35 and 55 minutes after pneumoperitoneum was significantly higher in VCV group than PCV group (*P*<0.05). Ventilation parameters are shown in Table-II. There were not any significant difference between two groups regarding PO_2_, PCO_2_ and pH, except 35 and 55 minutes after pneumoperitoneum. In mentioned times, patients in PCV group had significantly higher PO_2_ levels compared to VCV group. Intra and post-operative variables of blood gas analysis are shown in Table-III. Intra and post-operative hemodynamic variables of two groups are shown in Table-IV. After three hours, post anesthesia nasal oxygen and analgesic requirements were similar between two groups.

**Table-I:** Demographic characteristics of patients in two groups.

	*VCV*	*PCV*	*P-value*
Age	33.74±8.62	33.08±8.59	NS
Gender M/F	22/13	23/12	NS
BMI	33.07±1.13	33.79±1.23	NS
ASA class I/II	23/12	25/10	NS
HR	88.54±8.79	87.08±9.15	NS
MAP	91.08±6.70	92.22±6.73	NS
FEV1 (lit/s)	3.1 (1.7-5.1)	3.2 (1.8-5.2)	NS
FVC (lit)	3.4 (2.1-5.1)	3.5 (2.2-5.7)	NS
FEV1/FVC	84 (77-88)	85 (78-92)	NS
TLC	5.1 (3.1-7.4)	5.2 (3.2-7.3)	NS

M/F: male/female, BMI: body mass index, HR: heart rate, MAP: mean arterial pressure, FEV: forced expiratory volume, FVC: forced vital capacity, TLC: total lung capacity, VCV: volume controlled ventilation, PCV: pressure controlled ventilation.

**Table-II:** Ventilatory and pulmonary indices of patients in two groups.

	*VCV*	*PCV*	*P-value*
ETCO_2_ base	36.77±1.55	36.74±1.54	0.939
ETCO_2_15	36.82±1.52	36.74±1.46	0.813
ETCO_2_ 35	37.17±1.58	37.20±1.53	0.939
ETCO_2_ 55	37.22±1.51	37.14±1.45	0.810
RR 15	11.62±0.97	11.65±0.93	0.901
RR 35	12.2±0.83	11.57±0.94	0.004
RR 55	12.37±0.87	11.42±0.88	0.000
V_T_ 15	628±53.31	641±54.95	0.351
V_T_ 35	646.57±56.73	613.28±55.27	0.015
V_T_ 55	648.57±57.44	610.57±57.88	0.007
Peak P 15	20.17±4.74	20.91±3.91	0.478
Peak P 35	25.05±4.79	20.22±4.32	0.000
Peak P 55	24.57±4.97	19.74±4.50	0.000
Mean P 15	8.97±2.74	9.02±2.67	0.930
Mean P 35	9.14±3.05	9.28±2.35	0.827
Mean P 55	9.57±2.86	9.82±2.45	0.688
Plat P 15	18.25±4.12	18.88±4.31	0.536
Plat P 35	18.22±5.08	19.17±4.79	0.427
Plat P 55	18.62±5.16	19.22±5.41	0.637

ETCO2: end tidal CO_2_, RR: respiratory rate, Vt: tidal volume, Peak P: peak airway pressure, Mean P: mean airway pressure, Plat P: plateau pressure, VCV: volume controlled ventilation, PCV: pressure controlled ventilation.

**Table-III:** Arterial blood gas analysis of patients in two groups.

	*VCV*	*PCV*	*P-value*
pH base	7.40±0.021	7.40±0.024	0.756
pH 15	7.38±0.01	7.39±0.02	0.728
pH35	7.39±0.02	7.39±0.01	0.218
pH 55	7.39±0.02	7.38±0.01	0.200
pH post op	7.42±0.02	7.41±0.01	0.652
PCO_2_ base	41.11±1.81	41.18±1.84	0.876
PCO_2_15	41.43±2.15	41.31±2.03	0.818
PCO_2_ 35	42.62±2.19	42.34±1.85	0.578
PCO_2_ 55	42.68±2.34	42.38±2.43	0.608
PCO_2_ post op	44.54±2.43	44.31±3.35	0.231
SPO_2_ base	98.28±0.62	98.48±0.65	0.196
SPO_2_15	98.57±0.65	98.62±0.64	0.714
SPO_2_ 35	98.62±0.59	98.65±0.59	0.841
SPO_2_ 55	98.57±0.60	98.51±0.65	0.707
SPO_2_ post op	98.21±0.56	98.30±0.61	0.684
PaO_2_ base	90.06±7.44	90.08±6.69	0.989
PaO_2_15	201.68±9.76	200.60±9.69	0.644
PaO_2_ 35	197.67±9.71	206.20±9.95	0.001
PaO_2_ 55	194.67±9.42	207.26±9.97	0.000
PaO_2_ post op	95.01±8.45	94.91±7.63	0.741

VCV: volume controlled ventilation, PCV: pressure controlled ventilation.

**Table-IV:** Hemodynamic parameters of patients in two groups

		*VCV*	*PCV*	*P-value*
HR	Intra. Op	71.54±9.46	70.32±8.37	NS
	Post. Op	89.64±10.11	90.13±9.94	NS
DBP	Intra. Op	73.31±6.83	72.24±6.31	NS
	Post. Op	88.07±7.95	89.01±7.63	NS
SBP	Intra. Op	111.12±12.31	110.17±11.47	NS
	Post. Op	132.31±13.41	131.22±12.88	NS
MBP	Intra. Op	89.21±7.37	88.45±7.87	NS
	Post. Op	100.01±9.94	99.02±9.31	NS

HR: heart rate, DBP: diastolic blood pressure, SBP: systolic blood pressure, MBP: mean blood pressure, VCV: volume controlled ventilation, PCV: pressure controlled ventilation. (The numbers are mean±SD)

## DISCUSSION

The results of our study showed that lung dynamic indices and oxygenation parameters during laparoscopic cholecystectomy didn’t have significant difference between VCV and PCV groups except in 35 and 55 minutes after pneumoperitoneum. Decreased lung compliance in obese patients lowered FRC resulting in closure of small airways during ventilation and ventilation perfusion mismatches and hypoxemia. As anesthesia and pneumoperitoneum cause more decrease in these parameters, choosing the ideal ventilation mode which prevents ventilator induced lung injury and improves oxygenation is necessary to decrease morbidity and mortality.^3^,^13^

Boules et al. demonstrated that there was no significant difference between VCV and PCV regarding respiratory and oxygenation parameters which was similar to our study.^14^ Samantaray and Hemanth demonstrated similar results to our study especially in later phase of anesthesia.^15^ In this prospective study, 35 patients post cardiac surgery patients were randomized to receive PRVC or PCV. There was a steady and significant improvement in oxygenation index in both groups at the first and second hours of ventilation. However, the improvement in oxygenation index was more marked in PRVC at the second hour of ventilation owing to significant low mean air way pressure compared to the PCV group [PCV, 8.6(0.8); PRVC, 7.7(0.5), P = 0.001]. One explanation for this difference in parameters can be the fact that PCV results in higher mean airway pressure during time which leads to better oxygenation toward the end of anesthesia. During inspiratory phase, mean airway pressure determines the distribution of ventilation and recruitment of collapsed alveoli, and is an important factor for gas exchange. PCV may lead to volutrauma/atelectrauma, so it is better to use volume-guaranteed PCV especially in morbid obese patients undergoing laparoscopy which have high intra-abdominal pressure. Sen et al. showed that PCV compared to VCV was associated with lower peak and plateau pressures and better oxygenation indices in both prone and supine positions before and during pneumoperitoneum.^2^ Assad et al. showed that volume guaranteed PCV is better than VCV in patients who underwent surgical cholecystectomy in trendelenburg position regarding lower peak inspiratory pressure and greater dynamic compliance.^16^ A meta-analysis showed that there is no significant difference between two modes in obese patients undergoing surgery but there is some positive evidence regarding intra and postoperative effects of PEEP and because of concerns about the effect of PEEP we deleted its effect with setting equal PEEP in both groups.^17^ Another meta-analysis compared VCV with PCV during laparoscopic cholecystectomy and showed lower peak pressure and higher compliance and mean airway pressure with PCV mode. Subgroup analysis showed the same results with the morbid obese patients.^18^ Jiang et al. in another meta-analysis compared PCV and VCV in 1643 patients in different positions (supine, lateral and prone)and conditions (one lung ventilation and laparoscopy) and showed that VCV is associated with decreased oxygenation index and increased alveolar-arterial oxygen difference. Subgroup analysis showed the same results during laparoscopy in obese patients.^19^ As mentioned, PCV can increase mean airway pressure which with its effect on pleural pressure may increase hemodynamic instability especially during pneumoperitoneum. This is not shown in our study which may be due to the little difference in mean airway pressure between two groups. Two studies compared the hemodynamic characteristics of patients undergoing PCV and VCV during anesthesia and showed no significant difference between groups which was similar to our results.^20^,^21^ Contrary to these results, De Baerdemaeker et al. showed no advantages of PCV regarding gas exchange, pulmonary mechanics, and risk of barotraumas compared to VCV. They also showed that CO_2_ elimination is more effective with VCV compared to PCV which may be because of different minute ventilation.^22^

Most of the studies comparing the effects of VCV and PCV during anesthesia were not well-designed and did not explain when and how to use each mode. Benefits from PCV (lower work of breathing and patient comfort)usually comes from decelerating flow waveform and benefits from VCV are related to reducing volutrauma.^23^ PCV has no advantage compared to PCV if patient does not have spontaneous breathing especially if VCV uses decelerating flow. Therefore, it seems that we won’t have the complications of each mode if we use dual modes and it can result in better lung and oxygenation parameters, and consequently decrease the ventilator induced lung injury.

### Limitation of the study

The patients enrolled in our study didn’t have any cardiac or underlying pulmonary diseases which may alter clinical findings; so, our results may not be applicable to populations with underlying pulmonary or cardiac problems.

## CONCLUSION

Despite some beneficial effects regarding plateau and mean airway pressure with PCV, there is no significant clinical difference between VCV and PCV in obese patients undergoing laparoscopic cholecystectomy. It seems that using dual modes would be an ideal approach with lower complications.

### Authors’ Contribution

**RM and AM:** Performing study, study design, manuscript writing.

**VF and MM:** Performing study, collecting data.

**AI:** Manuscript writing and editing.

**SS:** Analysis of the study results, editing and reviewing the manuscript.

All authors have read and approved the manuscript.
